# Is directly observed tuberculosis treatment strategy patient-centered? A mixed method study in Addis Ababa, Ethiopia

**DOI:** 10.1371/journal.pone.0181205

**Published:** 2017-08-01

**Authors:** Belete Getahun, Zethu Zerish Nkosi

**Affiliations:** 1 University of South Africa, College of Human Sciences, Department of Health Studies, Pretoria, South Africa; 2 Addis Ababa University, College of Health Sciences, School of Public Health, Addis Ababa, Ethiopia; McGill University, CANADA

## Abstract

**Introduction:**

The directly observed treatment, short course (DOTS) strategy has been considered as an efficacious approach for better tuberculosis (TB) treatment adherence and outcome. However, its level of patient centerdness has not been studied and documented well. Hence, the study aimed to determine the level of patient centeredness’ of the DOTS.

**Method:**

The study used explanatory sequential mixed method design in Addis Ababa, Ethiopia. The study employed an interviewer-administered questionnaire with 601 patients with TB, focus group discussions with 23 TB experts, and telephonic-interview with 25 persons lost to follow-up from TB treatment. Descriptive and multivariable analyses carried out for the quantitative data while thematic analysis was used for the qualitative data.

**Result:**

Forty percent of patients with TB had not received patient-centered TB care (PC-TB care) with DOTS. Male gender (AOR = 0.45, 95% CI 0.3, 0.7), good communication (AOR = 3.2, 95%CI 1.6, 6.1), and health care providers as a treatment supporter (AOR = 3.4, 95% CI 2.1, 5.48) had significant associations with PC-TB care. All persons lost to follow-up and TB experts perceived that DOTS is merely patient-centered. The identified categories were patient preferences, treatment supporter choice, integration of DOTS with nutritional support, mental health, and transport services, provider’s commitment and communication skills.

**Conclusion:**

DOTS is limited to provide patient-centered TB care. Hence, DOTS needs a model that enhances effectiveness towards patient centeredness of TB care.

## Background

In the mid-1990s, World Health Organization (WHO) developed and recommended directly observed treatment, short course (DOTS) for the treatment of TB. The DOTS strategy encompasses political and administrative commitment, case detection primarily by microscopic examination of sputum of patients presented to health facilities, standardized short course chemotherapy given under direct observation, adequate supply of good quality drugs and systematic monitoring for every patient diagnosed [1]. The DOTS has been considered as a cornerstone and efficacious for better treatment adherence of TB control programme in developing countries [2–4]. Moreover, DOTS is perceived to be associated with decreased probability of acquiring and transmitting drug resistance [5], and improved treatment success rate [6].

However, DOTS has been criticized to pose an economic and social burden to patients and to the health facilities [7]. Its approach, particularly daily observation of patients with TB at health facilities while taking the treatment is exposing the majority of patients with TB for catastrophic cost especially in developing countries [8–11].

Patient-centered TB (PC-TB) care is associated with better treatment adherence, improved patient outcome and quality of life of patients with TB [12]. In cognizant of the PCC benefits, post 2015 TB care delivery approach recommends integrated PCC as a pillar for TB control activities together with other two pillars. These pillars are expected to support the TB control activities in making a world free of TB, zero death, disease and suffering due to TB in 2035 [13].

Although there is no universally accepted patient-centered care (PCC) model for TB treatment, different scholars proposed and tested different components of PCC as a PC-TB care model. Tested PCC components as PC-TB care were task shifting from health facilities to the community [14], treatment supporter choice provision either health care provider (HCP) or family member [15] and power sharing between the HCPs and patients with TB [16]. Task shifting to the community was equally effective and efficient as DOTS at health facilities [14]. In addition, task shifting to the community health workers improved access, service utilization and routine TB recording and reporting systems [17]. Provision of treatment supporter choices to the patient and empowering patients brought a significant improvement in TB treatment outcome and decreased internalized stigma [14]. However, the DOTS patient centeredness has not been studied well, particularly, using overarched model that considers a wide range of perceived factors for provision of PC-TB care. Therefore, the study aimed to determine the level of patient centeredness and associated factors with the DOTS strategy using WHO people centered health care policy framework in Addis Ababa Ethiopia.

## Materials and methods

### Setting

The study carried out in Addis Ababa, a capital city of Ethiopia, from September 2015 to November 2015. The Addis Ababa’s population size, administrative structure and its TB care provision system have been reported elsewhere in detail [18]. At the time of the study period, 121 health facilities were providing TB treatment with DOTS. The 121 health facilities were listed and categorized into government, private for profit and non-government not for profit. Among categorized health facilities, by rule of thumb, approximately 25% of the health facilities from each category was randomly selected by lottery method. A total of 30 study health facilities were selected (Table 1).

**Table 1:** Study sites by subcity, ownership and type.

Study site facility	Type of facility	Sub-city	Ownership
SS1	Health centre	Adisketema	Gov
SS2	Health centre	Adisketema	Gov
SS3	Health centre	Akakikality	Gov
SS4	Health centre	Arada	Gov
SS5	Health centre	Bole	Gov
SS6	Health centre	Bole	Gov
SS7	Health centre	Gulele	Gov
SS8	Health centre	Gulele	Gov
SS9	Health centre	Kirkos	Gov
SS10	Health centre	Kirkos	Gov
SS11	Health centre	Kolfe K	Gov
SS12	Health centre	Kolfe K	Gov
SS13	Health centre	Lideta	Gov
SS14	Health centre	Lideta	Gov
SS15	Health centre	Nifas L	Gov
SS16	Health centre	Nifas L	Gov
SS17	Health centre	Yeka	Gov
SS18	Health centre	Yeka	Gov
SS19	Hospital	Arada	Gov
SS20	Hospital	Kirkos	Gov
SS21	Health centre	Arada	Gov
SS22	Health centre	Bole	Gov
SS23	Health centre	Gullele	Gov
SS24	Clinic	Arada	NGO
SS25	Clinic	Lideta	Private
SS26	Hospital	Arada	Private
SS27	Hospital	Bole	Private
SS28	Hospital	Bole	Private
SS29	Hospital	Kirkos	Private
SS30	Hospital	Yeka	Private

GOV: Government, NGO: non-government for not profit.

Before starting data collection ethical clearance was obtained from Institutional Research and Higher Degrees committee from the University of South Africa. In addition, Addis Ababa City Administration, Health Bureau permitted to access the study subjects at health facilities. The data were collected after written consent obtained from each participant, each participant personal identifiers were not used and collected data were not shared with anybody except the research team. Interview was conducted in a way that did not deprive study participants privacy.

### Study design

The study used a sequential explanatory mixed design. In which quantitative and qualitative techniques were given equal priority to prevent the skewness of the response that may come only from patients with TB who were on follow-up of TB care in quantitative approach.

### Study population and data collection

The study included randomly selected 605 patients with TB who were on follow-up of TB treatment and 23 persons lost to follow-up from TB care and purposively selected 23 TB experts. The samples size, 605 patients with TB who were on follow-up of TB care, was determined based on single population proportion formula. The assumptions for determining the sample size were a) 50% of patients with TB expected to receive PC-TB care, b) 0.05 error allowance, c) 1.96 two-sided critical value for 95% confidence level and 0.05 level of perceived PC-TB care significance, d) 1.5 for design effect compensation and e) 5% contingency for non-response rate.

The WHO people centred health care policy framework adopted interviewer-administered questionnaire was used to determine DOTS patient centeredness for patients with TB who were in follow-up [19].

The interviewer-administered questionnaire was prepared in English then translated in to Amharic (the official and local language of Addis Ababa). The questionnaire had two parts: the socio demographic characteristics and 65 patient centeredness measuring items. The 65 items were categorised into four dimensions: TB Care Delivery System, Health Care Organization (HCO), HCP and patient, family and community dimension. All the 65 measuring items were measured by 5-point Likert scales ranging from strongly disagree to strongly agree. The scores were ranged from 1 to 5 to strongly disagree to strongly agree, respectively for positively worded items and inversely for negatively worded items. The questionnaire was administered by 10 Data Collectors who trained on the questionnaire and ethical principles of human subject involved researches. The data collection processes was supervised by the principal researcher and two supervisors.

Telephonic-interview with 25 randomly selected persons lost to follow-up from TB care and 3 focus group discussions (FGDs) with conveniently selected 23 TB experts were carried out. The telephonic-interview and FGDs were facilitated in Amharic based on prepared leading questions. The principal researcher carried out the telephonic-interviews while the principal researcher and research assistant facilitated the FGDs.

### Quantitative data analysis

The quantitative data were analyzed using Statistical Package for Social Science (SPSS) version 21.0 for Windows (Chicago, IL, USA). The variables described in frequency distribution, percentage, central tendency and dispersion such as standard deviation, range and confidence intervals. The overall and each dimension Likert scale measuring items Cronbach’s alpha were measured during pilot and actual data analysis (Table 2).

**Table 2:** Cronbach’s alpha value of patient centeredness measuring questionnaire.

Dimension	No of Items	Cronbach’s alpha value of pilot data	Cronbach’s alpha value of actual data
Patient, family and community dimension	21	0.82	0.84
HCP dimension	22	0.85	0.87
HCO dimension	9	0.76	0.76
Health care system dimension	13	0.88	0.88
**Overall centeredness**	**65**	**0.93**	**0.93**

To determine the proportion of patients with TB who received PC-TB care, the five Likert scales were adjusted into a 0-to-100 scale by utilizing a Likert’s transformation formula [20]. The respondents who score above the mean of adjusted score were considered as received PCC with DOTS.

Logistic regression was used to identify the subset of measured independent variables association with received PC-TB care. The independent variables were used based on direct response of the respondents, except average monthly family income. Average monthly family income dichotomized into two based on the mean family-income value. Those respondents who had above the mean of the average of monthly family income considered as “less-poor” whereas respondents who had below and equal to the mean of average monthly income regarded as “poor’’. To determine the presence of association between variables, p value, Chi-square and Adjusted Odd ratios (AOR) were used where appropriate [21].

### Qualitative data analysis

The qualitative data analysis was carried out by the researcher and a public health specialist, an expert in qualitative research methodology, independently to create codes, categories and identify themes. Collected field notes and audios in Amharic language were translated and transcribed verbatim into English by the researcher and research assistant. The collected data were compiled, transcribed and emerging ideas were listed without strict sequences. Then codes, categories and sub-categories for the listed ideas were created. Recoding was done when necessary. Drawing lines were used to relate the categories, then after themes were generated. Finally, a consensus meeting between the researcher and the public health specialist was held. In the meeting the categories and themes identified were compared, revised and then agreed themes were used as research findings for the study.

### Triangulation

Each set of data extracted using an interviewer-administered questionnaire, in-telephonic-interviews and FGDs were separately analyzed. The findings of each set of data were critically observed to assess either the set of findings is convergent or divergent and triangulated at an interpretive level to complement the weakness of one source of data over the other [22].

## Result

### Study groups compositions

The study included three set of study population groups: patients with TB who were attending for TB treatment, persons lost to follow-up from TB treatment and TB experts. Among 605 patients with TB, 601(99%) patients were consented to participate in the study. Of which 336 (56%) were male participants. Five hundred one (83%) were new patients with TB (Table 3).

**Table 3:** Gender and treatment category of patients with TB (N = 601).

Gender	Treatment category patients with TBs						Total *f* (%)
	New *f* (%)	Relapse *f* (%)	Treatment after failure *f* (%)	Return after default *f* (%)	Transfer-in *f* (%)	Other *f* (%)	
Female	221(83.4)	33(12.5)	2(0.8)	1(0.4)	5(1.9)	3(1.1)	265(100)
Male	280(83.3)	35(10.4)	10(3.0)	3(0.9)	8(2.4)	0(0)	336(100)
**Total**	**501(83.4)**	**68(11.3)**	**12(2.0)**	**4(0.7)**	**13(2.2)**	**3(0.5)**	**601(100)**

*f* = frequency; % = percentage

Twenty-one percent of patients with TB were 18-24 years of age, 36% were 35-44 years of age, 25% were 35-44 years old, 10% were 45-54 years old 5% were 55-64 years old and only 3% were above 65 years old. While among 23 TB experts, 35% of TB experts’ age were between 20-30 years, 13% were 31-40 years, 44% were 41-50 years and 7% were 51-60 years. The average monthly family income of patients with TB was $115 (SD = $97). The average monthly income across type of TB is described in Table 4.

**Table 4:** Average monthly family-income and type of TB (N = 546).

Average monthly family-income in USD	Type of TB				Total *f(%)*
	SmearPositive PTB f*(%)*	Smear Negative PTB *f(%)*	Extra PTB *f(%)*	MDR-TB *f(%)*	
9.40-47.00	78(42.9)	37(20.3)	55(30.2)	22(6.6)	182(100)
47.1-95.00	66(44.9)	35(23.8)	39(26.5)	7(4.8)	147(100)
95.01-143.0	33(40.7)	25(30.9)	19(23.5)	4(4.9)	81(100)
143.1-957.0	60(44.1)	32(23.5)	38(27.9)	6(4.4)	136(100)
**Total**	**237(43.4)**	**129(23.6)**	**151(27.7)**	**29(5.3)**	**546(100)**

USD: United State dollar; Figures are computed at Ethiopia average National Bank exchange rate of Birr 20.8979 to 1USD in October 2015. PTB: Pulmonary TB; MDR:Multi Drug Resistance TB

The majority 511 (85%) of the respondents were urban residents, of these 228 (46.6%) were smear (+) patients with PTB (Table 5).

**Table 5:** Place of residence and type of TB of the respondents (N = 601).

Residence	Type of TB				Total *f(%)*
	SmearPositive PTB *f(%)*	Smear Negative PTB *f(%)*	Extra pulmonary TB *f(%)*	MDR-TB *f(%)*	
Urban	228(44.6)	126(24.7)	132(25.8)	25(4.9)	511(100)
Rural	32(46.4)	17(24.6)	16(23.2)	4(5.8)	69(100)
Homeless*	3(14.3)	4(19)	11(52.4)	3(14.3)	21(100)
**Total**	**263(43.8)**	**147(24.5)**	**159(26.5)**	**32(5.3)**	**601(100)**

* Patients with TB who do not have formal house either in urban or rural

PTB: Pulmonary TB; MDR:Multi Drug Resistance TB.

Among 25 persons lost to follow-up, 15 (58%) were male. Median age of persons lost to follow-up was 30 (Range = 27 years). Among 19 fully responded persons lost to follow-up, 7 (37%) of them attended first cycle primary school (grade 1-4), 6 (32%) of them attended second cycle primary school (grade 5-8), 4 (21%) of them attended secondary school (grade 9-12) and 2 (10%) of them had no formal education.

Among 23 FGD participants, 15 (65%) were male and the least TB related work experiences of the participant was 2 years (Table 6).

**Table 6:** Gender, age and work experience of FGD participants (N = 23).

Variables	Category	*f* (%)
Sex	Male	15 (65.2)
	Female	8 (34.8)
Age in years	20-30	8 (34.8)
	31-40	3 (13.0)
	41-50	10 (43.5)
	51-60	2 (8.6)
TB-related work experience (in years)	2-4	8 (34.8)
	5-6	5 (21.7)
	7-8	6 (26.1)
	9-15	4 (17.4)

### Patient centeredness of DOTS for patients with TB

Patient, family and community concern dimension items mean score was 3.27 (SD = 0.59). Each measuring items frequency, mean score and SD are presented in Table 7.

**Table 7:** Patient, family and community concern dimension items mean scores (N = 601).

Items	Strongly disagree *f (%)*	Disagree *f (%)*	Neutral *f (%)*	Agree *f (%)*	Strongly agree *f (%)*	Mean (SD)
**Information provision about:**						
importance of treatment supporter	35 (5.8)	53 (8.8)	32 (5.3)	394 (65.6)	87 (14.5)	3.74 (1.00)
TB transmission, prevention and treatment	40 (6.7)	51 (8.5)	17 (2.8)	415 (69.1)	78 (13.0)	3.73 (1.01)
health condition prognosis	44 (7.3)	49 (8.2)	49 (8.2)	386 (64.2)	82 (13.6)	3.70 (1.01)
wrong practice about TB	55 (9.2)	75 (12.5)	49 (8.2)	349 (58.1)	73 (12.1)	3.51 (1.10)
**Keeping preference, HCPs accept your choice of**						
treatment supporter	99 (16.5)	131 (21.8)	107 (17.8)	221 (36.8)	43 (7.2)	2.96 (1.23)
drug collection time	195 (32.4)	227 (37.8)	43 (7.2)	136 (22.6)	0 (0)	2.19 (1.12)
where to take the treatment	498 (82.9)	103 (17.1)	0 (0)	0 (0)	0 (0)	1.17 (0.37)
**Recognition: taking**						
your consent during decisions	105 (17.5)	131 (21.8)	74 (12.3)	255 (42.4)	36 (6.0)	2.97 (1.25)
your role in account	108 (18.0)	129 (21.5)	70 (11.6)	265 (44.1)	29 (4.8)	2.96 (1.25)
part in treatment plan	107 (17.8)	162 (27.0)	92 (15.3)	215 (35.8)	25 (4.2)	2.81 (1.21)
**Improving capacity your self-management and self-care with:**						
counselling	38 (6.3)	53 (8.8)	27 (4.5)	417 (69.4)	66 (11.0)	3.69 (0.99)
health education	51 (8.5)	61 (10.1)	42 (7.0)	380 (63.2)	67 (11.1)	3.58 (1.08)
written, pictorial and audio-visual materials	98 (16.3)	69 (11.5)	42 (7.0)	315 (52.4)	77 (12.8)	3.33 (1.30)
**Your treatment supporter**						
know the transmission route, prevention and treatment of TB	40 (6.7)	40 (6.7)	20 (3.3)	374 (62.2)	126 (21.0)	3.84 (1.04)
observe while taking drugs	33 (5.5)	70 (11.6)	43 (7.2)	364 (60.6)	91 (15.1)	3.68 (1.04)
regularly communicate and discuss with you and HCP	43 (7.2)	102 (17.0)	48 (8.0)	341 (56.7)	67 (11.1)	3.64 (1.11)
**Family and friends involvement in TB care:**						
family discuss pros and cons about TB with you	58 (9.7)	118 (19.6)	48 (8.0)	293 (48.8)	84 (14.0)	3.77 (1.22)
friends discuss pros and cons about TB with you	45 (7.5)	49 (8.2)	54 (9.0)	358 (59.6)	95 (15.8)	3.68 (1.07)
families encouraged and allowed to participate	47 (7.8)	54 (9.0)	42 (7.0)	379 (63.1)	79 (13.1)	3.64 (1.06)
friends encouraged and allowed to participate	60 (10.0)	87 (14.5)	73 (12.1)	313 (52.1)	68 (11.3)	3.40 (1.16)
**Total Mean Score**						**3.27 (0.59)**

SD: standard deviation; *f*: frequency; %: percentage

The HCP concerned dimension mean score was 3.72 (SD = 0.49). The lowest mean score 2.68 (SD = 1.24) among the HCPs’ dimension measuring item was concern of HCPs to discuss on financial issue of patients with TB. The detail means score of each items are depicted in Table 8.

**Table 8:** Health Care Providers (HCPs) perspective items mean scores (N = 601).

Items	Strongly disagree *f (%)*	Disagree *f (%)*	Neutral *f (%)*	Agree *f (%)*	Strongly agree *f (%)*	Mean (SD)
**HCPs characteristics: HCPs are**						
committed	18 (3.0)	10 (1.7)	24 (4.0)	404 (67.2)	145 (24.1)	4.07 (0.78)
Accountable	17 (2.8)	12 (2.0)	26 (4.3)	403 (67.1)	143 (23.8)	4.06 (0.78
hones, respectful, compassionate and tolerance	18(3.0)	14 (2.3)	16 (2.7)	415 (69.1)	138 (23.0)	4.06 (0.78)
self-reflective	15 (2.5)	27 (4.5)	44 (7.3)	376 (62.6)	139 (23.1)	3.99 (0.84)
aware each other’s involvement	19 (3.2)	17 (2.8)	70 (11.6)	391 (65.1)	104 (17.3)	3.90 (0.82)
provide value, share information and responsibilities (each other’s)	20 (3.3)	24 (4.0)	82 (13.6)	360 (59.9)	115 (19.1)	3.87 (0.87)
**Considering a patient as a unique person: HCPs**						
respect your idea, culture and religion	11 (1.8)	18 (3.0)	23 (3.8)	411 (68.4)	137 (22.8)	4.07 (0.73)
understand your feelings	15 (2.5)	25 (4.2)	31 (5.2)	401 (66.7)	128 (21.3)	4.00 (0.81)
recognize and provides values	15 (2.5)	35 (5.8)	56 (9.3)	390 (64.9)	105 (17.5)	3.89 (0.84)
**HCPs and patient communication are**						
on prioritizing patient’s problem	13 (2.2)	20 (3.3)	20 (3.3)	443 (73.7)	105 (17.5)	4.01 (0.73)
free discussions	15 (2.5)	40 (6.7)	30 (5.0)	416 (69.2)	100 (16.6)	3.9 (0.83)
based on careful listening and understanding	15 (2.5)	33 (5.5)	30 (5.0)	459 (76.4)	64 (10.6)	3.87 (0.76)
built on mutual relationship	12 (2.0)	40 (6.7)	62 (10.3)	419 (69.7)	68 (11.3)	3.81 (0.79)
clear and summarised	31 (5.2)	41 (6.8)	54 (9.0)	405 (67.4)	70 (11.6)	3.73 (0.93)
regular and properly about medical condition	27 (4.5)	59 (9.8)	63 (10.5)	373 (62.1)	79 (13.1)	2.69 (0.97)
**Physical support, HCPs support in**						
keeping physical comfort	23 (3.8)	40 (6.7)	61 (10.1)	397 (66.1)	80 (13.3)	3.78 (0.89)
provision of assistance while feeling tire	23 (3.8)	39 (6.5)	55 (9.2)	398 (66.2)	86 (144.3)	3.80 (0.89)
**Emotional support, HCPs support to cope with**:						
relationships and mood changes	18 (3.0)	26 (4.3)	29 (4.8)	422 (70.2)	106 (17.6)	3.95 (0.81)
TB disease	28 (4.7)	18 (3.0)	18 (3)	432 (71.9)	105 (17.5)	3.94 (0.86)
cope with problems related to employment	59 (9.8)	81 (13.7)	95 (15.8)	283 (47.1)	83 (13.8)	3.41 (1.17)
**Biopsychosocial perspective *HCPs were concerned to discuss about*:**						
psychological status	91 (15.1)	94 (15.6)	57 (9.5)	313 (52.1)	46 (7.7)	3.21 (1.24)
family and social interactions	112 (18.6)	166 (27.6)	90 (15)	200 (33.3)	33 (5.5)	2.79 (1.23)
life history and development	103 (17.1)	192 (31.9)	97 (17.1)	176 (29,3)	32 (5.3)	2.73 (1.20)
financial issues	126 (21)	179 (29.8)	87 (14.5)	178 (29.6)	31 (5.2)	2.68 (1.24)
Total mean score						3.72 (.49)

The total mean score of HCO concerned dimension is 2.7 (SD = 0.42), frequency, percentage and mean score of each HCO concerned items are described in Table 9.

**Table 9:** HCOs concerned measuring items mean score (N = 601).

HCO	Strongly disagree *f (%)*	Disagree *f (%)*	Neutral *f (%)*	Agree *f (%)*	Strongly agree *f (%)*	Mean (SD)
**Ensuring access, effective and efficient coordination of care: HCO are**						
designed to keep comfort and safety	14 (2.3)	22 (3.7)	39 (6.5)	420 (69.9)	106 (17.6)	3.96 (0.77)
presence of reminder(how to cover mouth while coughing, hand and mouth care)	57 (9.5)	32 (5.3)	30 (5)	360 (59.9)	122 (20.3)	3.76 (1.2)
provision of health education at waiting rooms and premise	42 (7.0)	54 (9.0)	46 (7.7)	376 (62.6)	83 (13.8)	3.67 (1.04)
travelled longer distance to access health facility	86 (14.3)	132 (22)	32 (5.3)	276 (45.9)	75 (12.5)	3.20 (1.2)
**Establishing and strengthening multidisciplinary care teams:*Availability of patient support service*:**						
social support	547 (91)	16 (2.7)	22 (3.7)	14 (2.3)	2 (0.3)	3.32 (1.27)
food support	460 (76.5)	113 (18.8)	1 (0.2)	5 (0.8)	22 (3.7)	1.36 (0.85)
availing transport	566 (94.2)	11 (1.8)	2 (1.3)	19 (3.2)	3 (0.5)	1.13 (0.61)
spiritual support	102 (17)	59 (9.8)	36 (6.0)	351 (58.4)	52 (8.7)	1.04 (0.27)
**Total mean score**						**2.70(0.42)**

SD: standard deviation; *f*: frequency; %: percentage

DOTS service delivery system total mean score was 3.62 (SD = 0.52). The highest mean score in DOTS service delivery system on TB care service integration with HIV/ART services was 3.96 (SD = 0.92). The DOTS service delivery system measuring items frequency, percentage and mean scores are described in the Table 10.

**Table 10:** TB care delivery health system concerns measuring items (N = 601).

Items	Strongly disagree *f (%)*	Disagree *f (%)*	Neutral *f (%)*	Agree *f (%)*	Strongly agree *f (%)*	Mean (SD)
**Monitoring and addressing patient and community concerns about DOTS quality:**						
you are well transitioned from diagnosis to treatment	31 (5.2)	57 (9.5)	95 (15.8)	329 (54.7)	89 (14.8)	3.64 (1.01)
Well-arranged appointment for follow-up	22 (3.7)	26 (4.3)	36 (6.0)	401 (66.7)	115 (19.1)	3.93 (0.86)
Integrated TB care with HIV/ART	27 (4.5)	23 (3.8)	34 (5.7)	376 (62.6)	141 (23.5)	3.96 (0.92
HCPs mutual agreements about the care given to you	22 (3.7)	25 (4.2)	67 (11.1)	387 (64.4)	100 (16.6)	3.86 (0.86)
the name and contact details of the person in charge of TB care presented	68 (11.3)	65 (10.8)	49 (8.2)	303 (50.4)	116 (19.3)	3.55 (1.23)
confidentiality of clinical information were kept	19 (3.2)	19 (3.2)	48 (8.0)	341 (56.7)	174 (29)	3.60 (1.04)
**Teamwork and teambuilding**						
good inter-facility referral system	41 (6.8)	43 (7.2)	88 (14.6)	366 (60.9)	63 (10.5)	3.60 (1.00)
good intra-facility referral system	35 (5.8)	53 (8.8)	89 (14.8)	376 (62.6)	48 (8.0)	3.58 (0.96)
good collaboration among registration, laboratory, treatment and discharge services	32 (5.3)	44 (7.3)	86 (14.3	380 (63.2)	59 (9.8)	3.64 (0.94)
**Assisting people who experienced adverse events in the health system**						
assistance while you faced difficult situations in the TB care provision	36 (6)	74 (12.3)	97 (16.1)	342 (56.9)	51 (8.5)	3.49 (1.01)
compensation system for difficult situation, where appropriate	55 (9.2)	94 (15.6)	122 (20.3)	282 (46.9)	47 (7.7)	3.28 (1.1)
fixed contact persons is assigned for questions, problems and complaints	90 (15)	118 (19.6)	83 (13.8)	279 (46.4)	31 (5.2)	3.07 (1.2)
reassurance for inconveniences	41 (6.8)	56 (9.3)	76 (12.6)	372 (61.9)	56 (9.3)	3.57 (1.01)
**Total mean score**						**3.63 (0.52)**

SD: standard deviation; *f*: frequency; %: percentage

**Fig 1:**
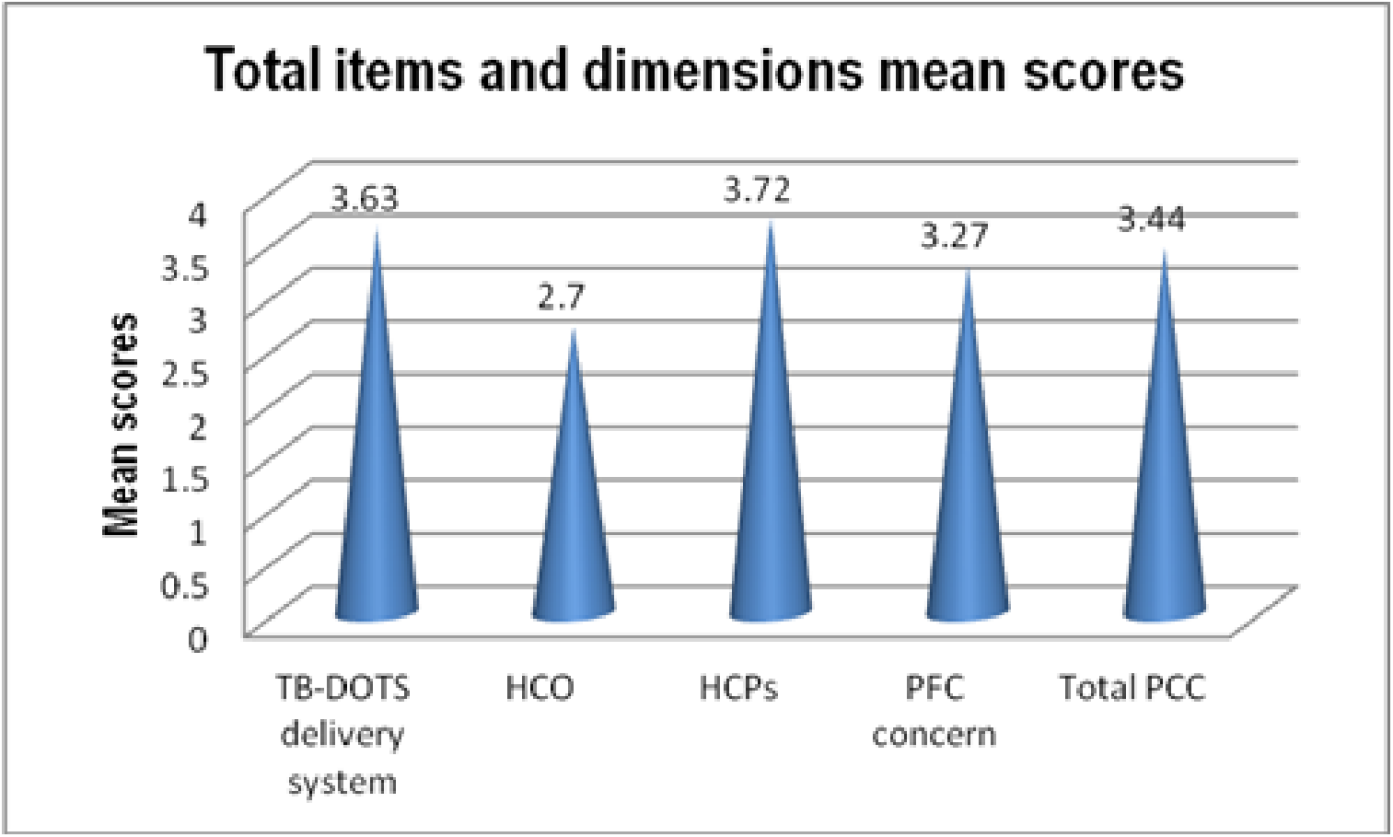
tif Mean score of PCC dimensions (N = 601).

The overall mean score of the 65 measuring items in the framework was 3.44 (SD = 0.44). The mean score of dimensions are dipcted in Fig 1.

Over all adjusted mean score of PCC with DOTS was 60 (SD = 11), 40% of the respondent’s score was below the mean, did not perceive as they received PC-TB care.

### Perceived PCC and Type of TB

From 242 respondents who did not perceive as they received PCC, 16 were patients with MDR TB (50% of -patients with MDR TB), 114 were smear positive PTB (47% of patients with smear positive PTB), 57 smear negative PTB (38% of patients with smear negative PTB) and 55 were EPTB (35% of extra pulmonary patients with TB). There was no significant association between PCC received with type of TB (p = 0.177).

### Perceived PCC and treatment category of patients with TB

**Fig 2:**
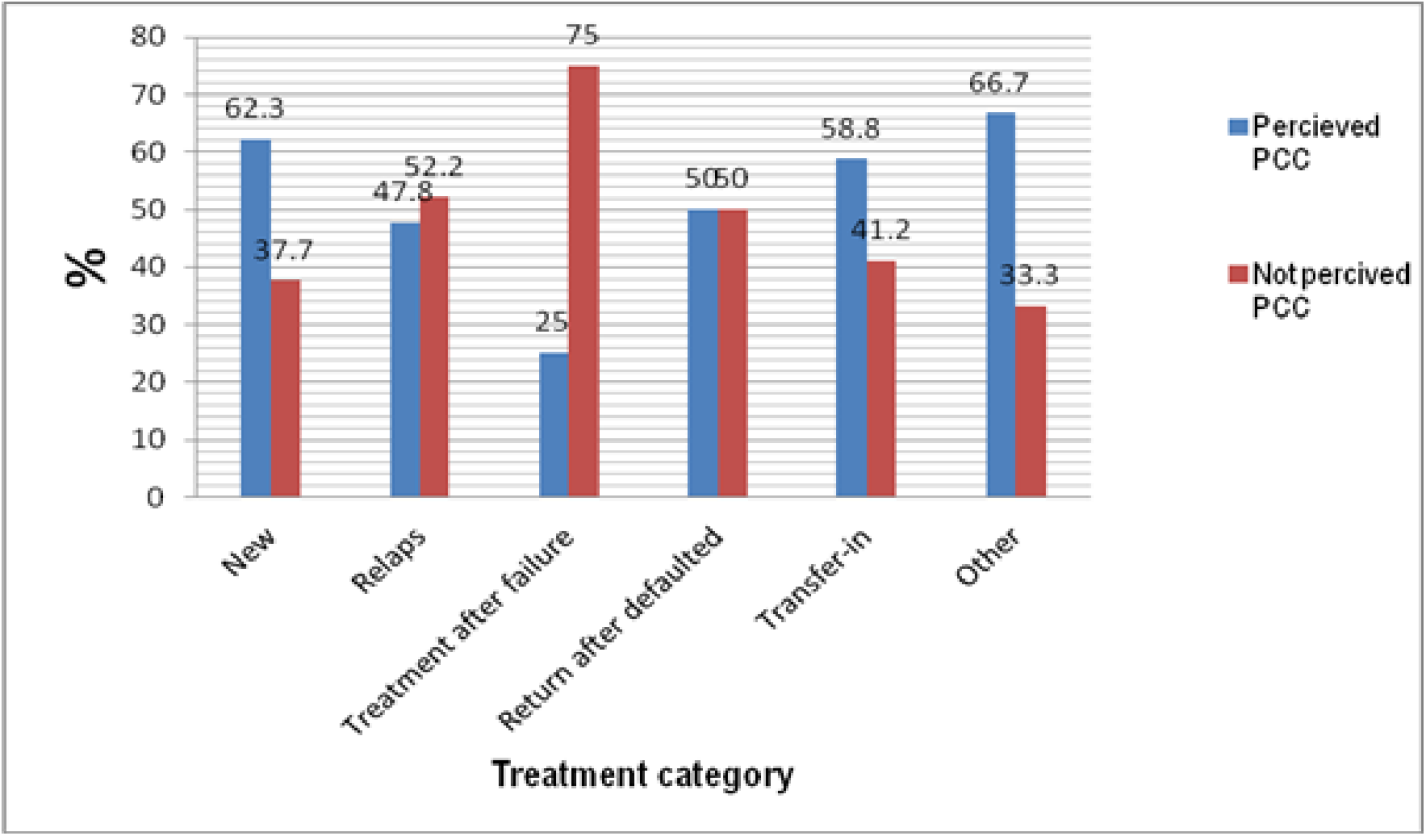
tif Perceived PCC and registration category.

Among 496 new patients with TB, 304 (61%) were received PCC with DOTS strategy. Whereas, as shown in Fig 2, among 12 respondents who were on treatment after treatment failure, only 3 (25%) perceived as the received PCC. Perceived PCC with DOTS and treatment category of the respondents had significant association (p = 0.019).

### Factors associated with PCC of DOTS

Gender, age, educational level, occupation, experience, presence of symptoms, treatment supporter type and perceived good communication had significant association with perceived PCC with DOTS. However, after adjusting the confounders in multiple logistic regression with the model fitness, Hosmer and Lemeshow test X^2 =^ 12.939, df = 8 and p = 0.114; gender, communication and type of treatment supporter type had association with perceived PCC with DOTS. The detail logistic regression analysis outcome is presented in Table 11.

**Table 11:** Logistic regression analysis of variables with perceive PCC with DOTS.

Variables		Perceived PCC		COR (95% CI)	P-value	AOR (95% CI)	P-value
		Yes	No				
Gender	Male	181	148	0.64 (0.46, 0.89)	0.009	0.45 (0.3, 0.7)	000
	Female	174	91	1.00		1.00	
Age	18-24	64	63	1.00		1.00	
	25-34	125	89	1.38 (0.89, 1.43)	0.15	1.1 (0.65, 1.8)	0.68
	35-44	103	4	2.4 (1.4, 3.8)	0.001*	1.8 (1.1, 3.9)	0.01
	45-54	39	19	2.0 (1.0, 3.8)	0.034	2.4 (1.1, 5.1)	0.06
	55-64	15	16	0.9 (0.4, 2.0)	0.84	0.9 (0.3, 2.2)	0.52
	≥65	9	9	0.98 (0.4, 3.6)	0.97	1.0 (0.3, 3.1)	0.42
Educational level	Diploma and above	46	95	1.5 (0.9, 2.9)	0.12	1.6 (0.75, 3.5)	0.20
	Secondary school (9-12)	70	122	1.3 (0.77, 2.3)	0.30	1.4 (0.73, 2.8)	0.41
	Primary school (5-8)	66	79	.92 (0.5, 1.6)	0.77	0.9 (0.5, 1.9)	0.75
	Primary school (1-4)	27	20	0.57 (0.2, 1.2)	0.14	0.7 (0.3, 1.7)	0.68
	No formal education	30	39	1.00		1.00	
Occupation	permanent employee	68	33	1.00		1.00	
	Self-employee	148	68	1.1 (0.6, 1.7)	0.83	1.0 (0.6, 1.8)	0.25
	Temporary employee	39	46	0.4 (0.2, 0.7)	0.003	0.4 (0.2, 0.8)	0.25
	House wife	40	34	0.5 (0.3, 1.0)	0.075	0.4 (0.17, 0.8)	0.20
	Unemployed	43	40	0.5 (0.2, 0.94)	0.03	0.58 (0.3, 1.1)	0.95
	Pensioner	12	11	0.53 (0.21, 1.33)	0.17	0.7 (0.2, 2.0)	0.96
	Student	5	7	0.35 (0.1, 1.1)	0.089	0.4 (0.09, 1.5)	0.86
Average monthly income	Poor	186	141	1.5 (1.07, 2.2)	0.01	1.35 (0.9, 2.0)	0.16
	Less poor	142	70	1.00		1.00	
Experience	Yes	92	84	0.65 (0.45, 0.92)	0.016	0.76 (0.5, 1.1)	0.18
	No	263	155	1.00		1.00	
Good communicate-on with HCPs	Yes	338	199	4 (2.2, 7.20)	000	3.2 (1.6, 6.1)	000
	No	17	40	1.00		1.00	
Treatment supporter	HCP	294	87	2.7 (1.8, 4.0)	000	3.4 (2.1, 5.48)	000
	Family	61	152	1.00			
Presence of TB symptoms	Yes	96	90	0.61 (0.43, 0.87)	0.006	0.69 (0.45, 1.0)	0.07
	No	259	149	1.00			

HCP: Health care providers; 1.00: reference category; COR: Crude Odds Ratio; AOR: Adjusted Odds Ratio; CI: confidence interval

*Fisher exact test

### TB experts’ perception of DOTS patient centeredness

TB experts view on patient centeredness of DOTS was related with patient preferences, treatment supporter and DOTS delivery system.

#### Patient preferences

Keeping preferences of patients with TB repeatedly raised issues in the discussion. It was declared as keeping preference of patients with TB existed only in “principle” and depends on HCPs personal inspiration. In principle, after diagnosed patients as they have TB, provision of place of choices for patients with TB to decide where they can follow their treatments is stated. However, the sturdy advice to patients to choose a nearby health facility rather than which they prefer is highly practiced. The TB experts explained the provision of choices where to follow the treatment as:

“Soon after a patient is diagnosed for TB, the patient is asked for a place of preference where to follow a treatment.”

However, the TB experts mentioned that patients with TB prefer to go to well-known facilities, which have already treated a lot of patients before, rather than going to the nearby health facility. Selecting famous health facilities were reported to cause work imbalance among district health centres. As a result, HCPs insist patients with TB not to select such facilities. The TB experts explained that the patients with TB case load imbalance occurrence across the facilities and influence to choose as:

“Some district health centers are bearing high number of patients with TB to provide TB treatment.”

TB experts explained that, although time adjustment have been existed in health centers where there are highly motivated HCPs are practicing, starting DOTS service provision early in the morning at 7:30 am, earlier than an official starting working hour of Ethiopia, was helpful toward keeping time preferences of patients with TB. The time adjustment enables some of patients with TB to take-in their TB drugs and go to their work. Besides, with HCPs personal initiation, provision of TB treatment at home bases for seriously ill patients with TB was reflected in the discussion. The verbatim explanation of extra motivated HCPs support to keep patients with TB preferences in the discussion was as:

“There are highly motivated HCPs who start TB treatment provision early in the morning and deliver the treatment at home in personal initiation.”

TB experts mentioned that there were many preference requests and change of preference of patients with TB through time. Patients with TB preference usually change from what they have preferred primarily, after two weeks or so of treatment time. As a result, TB experts reflected that keeping all the preferences of patients with TB is difficult with the current TB-DOTS service delivery direction. The following sentence was forwarded about keeping the preferences of patients with TB as:

“Considering all patients’ preference throughout the entire process of treatment will jeopardize the applicability of the DOTS service.”

#### Treatment supporter

TB experts explained that the treatment supporter can be a family member of patients with TB or anybody who is living in close relationship with the patients. The patients with TB usually get counselled on the importance of treatment supporter and obliged to bring a treatment supporter before the start of treatment. However, some patients with TB get difficulties to choose and bring the treatment supporter and are obliged to bring a person that may not support them at all. The TB experts explained that the difficulty of bringing treatment supporter as:

“Some patients bring people that they do not know, who later fail to show up.”

The treatment supporters’ role is to observe the patients with TB while taking in the drugs and reports to the HCPs about the adherence level of the patient. The treatment supporters are also expected to come every week with the patient, for whom they are accounted for, to communicate with the HCPs based on the checklist (treatment supporter card). The roles of the treatment supporter were in line with the recommendation of TB treatment guideline of Ethiopia [23]. However, the roles of treatment supporters were mentioned as it is not feasible in practice. The following verbatim sentences were forwards by the TB experts during the discussion as:

“At the end of the week the patients just tick all the days on treatment supporter card by themselves even without taking the pills. Let alone the treatment supporters, the patients themselves sometimes have to go and ask for the pills from other patients as they could not make it to the health institution.’’

The TB experts also agreed that using the treatment supporter and treatment supporter cards may not objectively indicate whether the patients with TB were adherent with TB treatments. As long as daily record of treatment supporter card requires daily observation of the patient, treatment supporter may rely on and trust the patients information and fill the treatment supporter card. The participants explained the treatment supporter roles and treatment supporter card weak contribution for adherence with TB treatment by the TB expert as:

“I don’t think treatment supporter and treatment supporter card are working well.”

#### DOTS service delivery system

The TB experts explained that at the initiation of the treatment process patient discusses with HCPs to start the treatment follow-up as in line with the principle of PCC. The discussion includes how long the treatment takes, and do’s and do not’s of the treatment. Furthermore, information provision and counselling are integral part of the treatment initiation process with DOTS. As a result, the patient reach to informed decision; and most patients with TB agree to follow their treatment at health facility at which their TB was identified. If they do not need to follow, referral will be written for those who choose other facility. The following sentences were forwarded to compare DOTS delivery system with PCC by the TB experts as:

“Obviously the first 15 days are totally patient-centred.”

“In general DOTS contain some components of a PCC because patients agree on all the preconditions of the treatment at the start and may tell you that from where they are and whether they cannot pursue the treatment anymore at diagnosed facility. If he/she cannot follow at diagnosed facility, referral paper will be written to another clinic near by the patient’s destination and choice. However, the rest of the process is more of a guided procedure, not focused on patient’s need.”

The TB experts explained that the principle of daily observation of patients at health facility in DOTS service delivery is so difficult especially after they started the follow-up, usually after two weeks or so. In addition, the TB experts mentioned that the patients with TB, in contrary to the DOTS principle, usually request to follow their treatment by themselves rather than coming on a daily basis after taking sometime. The TB experts explained how daily observation is difficult with respect to patients with TB as follow:

“Usually patients are seen when they exhaust to visit the health centers on a daily basis.”

The DOTS delivery system is inflexible to the patients’ preference and needs. Hence, the TB experts regarded the DOTS as it is not fully patient-centred. The TB experts explained the DOTS patient centeredness’ verbatim as:

“We cannot say it is entirely patient-centred.”

### Persons lost to follow-up’s perception on DOTS patient centeredness

In the telephonic-interview with persons lost to follow-up three categories were identified. Identified categories were DOTS delivery system, DOTS service integration and HCP perspectives.

#### DOTS delivery system

Persons lost to follow-up from TB treatment reported that information provision, respect and value provision to patients with TB were reported as less emphasised components in DOTS service delivery.

Information provision

Information provided to person lost to follow-up about TB during TB care delivery was very limited and lacked continuity. The information provision about TB at TB care delivery point was expressed by the respondents as:

“I have been informed about TB at the first day of the treatment, and then after, no one talked about it and you just swallow the drug and go back.”

“When I was diagnosed as I have TB, a lot of information about TB was given to me but I forget them since I was in stress by thinking about the illness.”

Respect and value

The lost to follow-up persons reported that respect and value are not provided during diagnosis and treatment process. However, they explained that they deserve respect and value. The value and the respect might start from simple recognition of the patient as a person who gives much attention about his health and family, and of course as have many responsibilities in other sector. The concern of the respect and values were reflected by the respondents as:

“Do not remind me! One day I had to go as early as possible and told him about ........ but he was treating me as I cannot tell you how.”

Another respondent explained as:

“It is hazy to speak about respect and value; sometimes give you much respect another day almost may throw your drug through a window.”

#### DOTS delivery integration

As the lost to follow-up persons explained the integrated service with TB care was only HIV diagnosis services. Most of the respondents mentioned that as they received HIV testing service. Whereas the respondents strongly suggested that availing other services such as nutrition support, transport and mental health service to be integrated. The persons lost to follow-up explained DOTS service integration and required integration of other services as follows:

“My blood tested for HIV infection, while I was following the TB treatment.”

“May be I would not default if I had transport availability to go daily.”

“I got HIV testing service but I would love there was nutrition support for us.”

#### Health care providers (HCPs) related

Some of the persons lost to follow-up explained that the HCPs were not devoted to provide TB care service. In contrary, some of the respondents explained that the HCPs were so keen. The disagreement views of lost to follow-up persons explained as:

“The HCP started writing while I was talking with, even was not willing to see my face and respond.”

Whereas others persons lost to follow-up explained that the HCPs’ compassionated care and support as:

“He, the HCP, was so kind while I was tired; even he was the one who holds me up to get into the car at the first week of the treatment.”

The majority of the telephonic-interview respondents agreed on less commitment of HCPs to work with patients with TB. The lost to follow-up persons explained that HCPs commitment and psychological support to patients with TB was not consistent among HCPs and across health institutions. The respondents put the level of commitment at two extremes. The extremes are range from holding up the weak patient to getting into the car to turning the face while the patients with TB request or raise an issue. These extremes were explained by a respondent as:

“I remember that, the HCP gave me my drugs in the car, almost 50 metres far from TB room.”

“The HCP was waiting for me until I really combat on to TB room window let alone provision of the drug to a place where I was.”

## Discussion

The study used overarched WHO health care policy framework to determine the level of patient centeredness of DOTS. As a result, the study indicates that, although feeling across dimensions was not similar, overall perceived PC-TB care is 60% among patients with TB who were on follow-up. Apart from this, none of the lost to follow-up persons perceived as they received PC-TB care. In addition, DOTS is rarely patient-centered in the view of TB experts’. However, in TB control strategy provision of PC-TB care was conceptualized at the introduction of DOTS [24], emphasized as a required component in stop TB strategy (2006-2015) and it is one of the core pillars to end TB epidemic [25].

The PCC focuses on considering patient’s point of view, situations on decision-making process with the patient [26], empowering people with TB and communities, social support programs, communication and partnership between health sectors and community [27]. The study shows that HCO’s dimension, particularly with regard to establishing and strengthening multi-disciplinary TB care teams to patients with TB is limited. However, evidence [26] stated that coordinated multi-disciplinary care services are starting position for delivery of PCC and it helps to avail the health care service in reduced cost. The availability of allied health care services to patients with TB are imperative for patients not only to avail PCC but also to provide health care services with affordable cost [27], apart from this, the study shows that the availability of allied services for patients with TB are not well integrated with DOTS especially nutritional, spiritual and social supports.

Similar with other scholars [28–30], the study shows significant association between feeling of patients as have good communication with HCPs and patient centeredness of DOTS. Patients with TB who feel as they have good communication with HCPs are more likely to receive PCC (AOR = 3.2, 95%CI 1.6, 6.1). Unlike other studies [28–30], in this study experience of using health care services does not show significant association with DOTS patient centeredness. In this study perceived PC-TB care received is significantly different between gender; males are less likely to feel as they received PCC [AOR = 0.45, 95% CI 0.3, 0.7] while type of TB, level of education and expectation of patients with TB did not show significant difference to PC-TB care.

Similar with Ahmed et al [31] and Constand et al [32], the highest mean score reported by patients with TB was prioritizing patients concern and communication of HCPs with patients. Consistently numerous evidence [19, 33, 34] stated that keeping preference of patients with TB is a key component to PC-TB care. However, although the TB treatment guideline of Ethiopia clearly put provision of choices to patients with TB where to follow the treatment either at home, workplace or health facility is possible [23], patients with TB were following their treatment only at health facilities against many preference request and dynamic need of patients’ preferences.

One of the unique feature of DOTS compared to many other disease control strategies is the requirement of treatment supporter who watches and witnesses whether the patient takes in the drug or not and to communicate with HCPs about the patients adherence level accounted for [23]. However, the study identifies that none of treatment supporter, either family or friend, has regular communication with HCPs about the patients with TB they were accounted for. Despite the fact, all patients with TB were obliged to take an individual, who is in charge of them, to health facility to start a treatment. Similar with report in Uganda [35], DOTS care delivery system in this study is tied with inflexibility to get referral paper once after treatment started. However, taking into account the patients' preferences during referral is essential to address access barriers to treatment adherence and improve treatment outcome. In addition, supplementing the referral system with feedback from recipient facility to referral facility is required to assess and trace barriers related to referral system [36].

Furthermore, the obligation and detention of patients with TB to take the treatment at home or in the community in the name of “treatment adherence” may lead to the question of “human rights” issue and may reflect a violation of right to health as stated in Article 12.1 – of the International Covenants on economic, social and cultural rights. The extended right not only to timely and appropriate health care but also to the underlying determinants of international health [37]. Rather providing quality and accessible community-based DOTS, or use of mobile-health technologies may replace the role of treatment supporters and reduce lost to follow-up and non-adherent patients with TB [38].

The community and individual patient’s level of knowledge about TB is a suggested requirement to reinforce the PCC. In addition, it is one of the limiting factors to enhance PCC [39–42]. Despite the fact, the study shows that weak provision of information about TB particularly after the patient started TB treatment is pertinent with DOTS. Consequently, this may hinder to follow the full course of treatment, patient involvement and informed decision [34].

The study was conducted at governmental and non-governmental health institutions that have been implementing DOTS in Addis Ababa, Ethiopia using overarched framework in quantitative and qualitative approaches. The study included different study groups and described the view of these groups’ perceived patient centeredness of DOTS. In addition, the outcome of the study can be generalized to different similar regions and countries at which DOTS is being implemented. However, the use of cross-sectional design to investigate patient centeredness’ of DOTS to patients with TB at a point in time may limits the degree to which causal inferences and generalizations.

## Conclusion

DOTS is limited to provide comprehensive PC-TB care even if it has fewer components of PCC for patients with TB particularly at the start of the TB treatment. DOTS lack to include many of PCC components such as keeping patients’ preferences and treatment supporter choice, provision with respect and value of patient with TB, and integration of allied services such as adequate information provision and counselling, nutritional support, mental health, and transport services. Further, it requires HCPs’ commitment, communication skill and strong support to the patient to cope with TB. Hence, a PC-TB care model that considers the important components to provide PC-TB care for patients with TB is required.

## Supporting information

S1 File TB patient questionnaire, FGD and telephone guides. (DOCX)

## Abbreviations

DOT: Directly Observed Treatment
DOTS: Directly Observed Treatment,Short course
EFMOH: Ethiopian Federal Ministry of Health
EPTB: Extra pulmonary tuberculosis
FGD: Focused group discussion
HCP: Health care provider
HCO: Health Care Organization
MDR-TB: Multi Drug Resistance TB
PCC: Patient-cenred care
PC-TB: care, Patient- centred TB care
PTB: Pulmonary TB
TB: Tuberculosis
UNISA: University of South Africa
WHO: World Health Organization
