# Supplementary material for: Is directly observed tuberculosis treatment strategy patient-centered? A mixed method study in Addis Ababa, Ethiopia
Source: PLoS One. 2017 Aug 1;12(8):e0181205. doi: 10.1371/journal.pone.0181205 (PMC5538709; doi:10.1371/journal.pone.0181205)
Supplement: S1 File — (DOCX) [file pone.0181205.s001.docx]

## English version of TB patients questionnaire

**Evaluation of directly observed tuberculosis treatment strategy in Ethiopia: patient centeredness and satisfaction’ questionnaire**

**Part I. Instruction**

1. Before collecting any information, the letter of ethical clearance and cooperation letter should be presented to the head/representative of the facility and get permission.
2. Before conducting interview the below study information sheet must be read to sure the respondents acquire the required knowledge whether to participate in interview or not. In addition, get the participant consent.
3. For all responses, it should be recorded what the respondent exactly wanted to say.
4. All the close-ended questions should be chosen and circled from the given choices unless and otherwise the response is different from listed choices.
5. If the response is different from the listed choices in close-ended questions, the response should be put in other and must be specified.
6. For open-ended questions, the response should be written in the form that have provided in the bracket at the end of each questions.
7. If the respondent has general comment it should be written at the end of questions on provided space.

**Part II. Study information sheet**

Greetings, my name is ________________and I have been assigned to collect data for the study, which is conducted by Mr Belete Getahun on Evaluation of DOTS strategy, Its TB patient centeredness and satisfaction in Addis Ababa. Today I would like to talk with you and gather information by prepared questionnaire about your experiences on DOTS program in Addis Ababa as one of the components of this study. We are evaluating DOTS strategy with respect to TB patient centeredness and satisfaction in order to capture lessons that can be used in future interventions. This study ethically has been approved by UNISAethical review board and has permission from Addis Ababa city Administration Health Bureau. It takes approximately 20 minutes. Though it seems long time, the study helps to improve the programme by generating the model which helps to improve patient satisfaction and centeredness. Your name will not be asked and unique identification is not required. Your response will be kept confidential, only be shared for research team. You do not have to discuss issues that you do not want. If you want to withdraw from the study any time during the discussion process, you will not be obliged to continue or give reasons for doing so. If you refuse or not, will not have any consequences on you and the services provided to you.

Do you have any question which is not clear?

Also if you have any question or anything that is not clear you can directly contact principal investigator of this study, Belete Getahun, by phone no 0911602272 or e-mail: [bgetahun150@gmail.com](mailto:bgetahun150@gmail.com)

I would like to appreciate your help in responding to this interview.

If you are clear with the information provided and agree to participate please sign on the consent form.

**Part III**

**Study Participant Consent Form**

I, the undersigned individual am oriented about the objective of the study. I have been informed that all of my information will be kept confidential and used solely for this study. In addition, I have been well informed that my name will not be asked and unique identification is not required. I have the right not to discuss issues that I do not want. If I want to withdraw from the discussion I will not be obliged to continue or give reasons for doing so. Nonetheless, my agreement to participate in this study is with the assumption that, the information that I provide during the discussion will help greatly to evaluate DOTS strategy and improve its centeredness’ and satisfaction of TB patients.

Date ________________ signature____________________________

**Part IV. Questions**

| **Se. no** | | **Measurement Items** | | **Response** | |
| --- | --- | --- | --- | --- | --- |
|  | | **General characteristics** | |  | |
| **1.** | | Gender of the respondent | | 1. Male 2. Female | |
| **2.** | | Age | | 1. 18 - 24 2. 25 -34 3. 35 – 44 4. 45 – 54 5. 55- 64 6. 65 and above | |
| **3** | | Marital status | | 1. Married 2. Single (Never married) 3. Separated 4. Divorced 5. Widowed 6. Cohabiting | |
| **4** | | Family size? | | _______ (including the respondent) | |
| **5** | | Residence | | 1. Urban 2. Urban slum 3. Rural 4. Homeless 5. Other (specify) | |
| **6** | | Highest educational level | | 1. Diploma and above 2. Preparatory 3. Secondary school 4. Primary school 5. No formal education | |
| **7** | | Occupation | | 1. Permanent employee 2. Self employee 3. Temporary employee 4. Unemployed 5. Pensioner | |
| **8** | | Average household/family monthly income | | 1 ___________ 2. No response | |
| **9** | | Religion | | 1. Orthodox 2. Protestant 3. Catholic 4. Muslim 5. No religion 6. Other | |
| **10** | | Ethnic group | | 1. Oromo 2. Amhara 3. Tigre 4. Gurage 5. Other (specify) | |
| **11** | | Did you receive health care service in this facility before this illness? | | 1. Yes 2. No | |
| **12** | | If yes, how many days did you visit the health institution before you diagnosed as you have TB? | | ______________ | |
| **13** | | Type of TB | | 1. Pulmonary positive TB 2. Pulmonary Negative TB 3. Extra Pulmonary TB 4. MDR-TB | |
| **14** | | Treatment category/registration group (please see treatment follow up card) | | 1. New 2. Relapse 3. Treatment after Failure 4. Return after Defaulted 5. Transfer in 6. Other(specify) | |
| **15** | | When did you start TB treatment? | | (DD/MM/YYYY) | |
| **16** | | Do you have TB symptoms now? | | 1. Yes 2. No | |
| **17** | | Total planed treatment duration? | | 1. Six months 2. Eight months 3. Other (specify)__________ | |
| **18** | | Currently how you are collecting your TB drugs | | 1. Daily 2. Weekly   3. Monthly 4. Other | |
| **19** | | How much you pay for transport per day in ETB? (if there are care giver include all costs) | | ______________ | |
| **20** | | Did you expect to come and collect the drugs every day, week or months for this much time? | | 1. Yes 2. No | |
| **21** | | Do you think that you and HCPs have good communication? | | 1. Yes 2. No | |
| **22** | | Who is your treatment supporter? | | 1. HCP 2. Family member 3. Health extension worker 4. Friend 5. Volunteer 6. Other (specify) | |
| 1. **Centeredness of TB care provided**   **1= Strongly disagree 2. Disagree 3. Indecisive 4. Agree 5. Strongly agree** | | | | | |
| **Patient, family and community** | | | | | |
| **1** | | | **Patient empowerment and involvement** | |  |
| **1.1** | | | **Information provision** | |  |
|  | | | *The HCPs gave you enough information about:* | |  |
| **1.1.1** | | | route of TB transmission, prevention and treatment | | **1 2 3 4 5** |
| **1.1.2** | | | importance of treatment supporter | | **1 2 3 4 5** |
| **1.1.3** | | | prognosis of your health condition | | **1 2 3 4 5** |
| **1.1.4** | | | what is wrong practice with TB patients with regard to treatment | | **1 2 3 4 5** |
|  | | |  | |  |
| **1.2** | | | **Keeping of preference** | |  |
|  | | | *HCPs accept your:* | |  |
| **1.2.1** | | | place of choices where to take the treatment such as at home, work place or health facility. | | **1 2 3 4 5** |
| **1.2.2** | | | TB drug collection time | | **1 2 3 4 5** |
| **1.2.3** | | | gave you a chance to your own treatment supporter | | **1 2 3 4 5** |
|  | | |  | |  |
| **1.3** | | | **Recognition** | |  |
| **1.3.1** | | | Took part in treatment plan | | **1 2 3 4 5** |
| **1.3.2** | | | Your consent was taken and considered during decisions | | **1 2 3 4 5** |
| **1.3.3** | | | Your role in the care process were duly acknowledged | | **1 2 3 4 5** |
|  | | |  | |  |
| **2** | | | **Improving capacity for self-management and self-care** | |  |
|  | | | *HCPs provide you:* | |  |
| **2.1** | | | self care TB management with health education | | **1 2 3 4 5** |
| **2.2** | | | counselling about how to care yourself with TB care and its treatment | | **1 2 3 4 5** |
| **2.3** | | | Written, pictorial, audiovisual material about TB care and its treatment | | **1 2 3 4 5** |
|  | | |  | |  |
| **3** | | | **Treatment supporter** | |  |
|  | | | *Your Treatment supporter:* | |  |
| **3.1** | | | know the transmission route, prevention and treatment of TB | | **1 2 3 4 5** |
| **3.2** | | | always observe while you take drugs | | **1 2 3 4 5** |
| **3.3** | | | communicate regularly with the HCPs about your treatment ( if not HCP) | | **1 2 3 4 5** |
| **3.4** | | | communicate and discuses with you regularly about TB treatment | | **1 2 3 4 5** |
|  | | |  | |  |
| **4** | | | **Family and friends involvement** | |  |
| **4.1** | | | Families were encouraged and allowed to participate in your TB care activities | | **1 2 3 4 5** |
| **4.2** | | | Friends were encouraged and allowed to participate in your TB care activities | | **1 2 3 4 5** |
| **4.3** | | | Pros and cons of TB transmission, treatment and care were discussed with friends | | **1 2 3 4 5** |
| **4.4** | | | Pros and cons of TB transmission, treatment and care were discussed with families | | **1 2 3 4 5** |
|  | | |  | |  |
| **Health care provider** | | | | | |
|  |  | | | |  |
| **1** | **Characteristics of HCPs** | | | |  |
|  | *HCPs are:* | | | |  |
| **1.1** | honest, respectful, compassionate and tolerant | | | | **1 2 3 4 5** |
| **1.2** | self-reflective | | | | **1 2 3 4 5** |
| 1.3 | accountable for TB patients | | | | **1 2 3 4 5** |
| **1.4** | committed to provide TB care | | | | **1 2 3 4 5** |
| **1.5** | are aware of each other’s involvement | | | | **1 2 3 4 5** |
| **1.6** | provide value, share information and responsibilities(each others) | | | | **1 2 3 4 5** |
|  |  | | | |  |
| **2.** | **Patient as a unique Person** | | | |  |
|  | *Mostly, HCPs:* | | | |  |
| **2.1** | respect your idea, culture and religion | | | | **1 2 3 4 5** |
| **2.2** | recognize and provides values | | | | **1 2 3 4 5** |
| **2.3** | understand your feelings | | | | **1 2 3 4 5** |
|  |  | | | |  |
| **3** | **HCPs and- patient communication** | | | |  |
|  | *HCPs and your communications are:* | | | |  |
| **3.1** | on prioritising your problem | | | | **1 2 3 4 5** |
| **3.2** | free discussions | | | | **1 2 3 4 5** |
| **3.3** | based on are careful listening and understanding | | | | **1 2 3 4 5** |
| **3.4** | meant to build mutual relationship | | | | **1 2 3 4 5** |
| **3.5** | get clear and summarised information about your illness/medical condition | | | | **1 2 3 4 5** |
| **3.6** | deal about your medical condition regularly and appropriately | | | | **1 2 3 4 5** |
|  |  | | | |  |
| **4** | **Physical support** | | | |  |
|  | *HCPs support:* | | | |  |
| **4.1** | to keep your physical comfort | | | | **1 2 3 4 5** |
| **4.2** | provide assistance while you feel tired | | | | **1 2 3 4 5** |
|  |  | | | |  |
| **5** | **Emotional support** | | | |  |
|  | *The HCPs emotionally support:* | | | |  |
| **5.1** | to cope with the TB | | | | **1 2 3 4 5** |
| **5.2** | to cope with relationship and mood changes | | | | **1 2 3 4 5** |
| **5.3** | to cope with problems related to employment | | | | **1 2 3 4 5** |
|  |  | | | |  |
| **6** | **Biopsychosocial perspective** | | | |  |
|  | *HCPs are concerned:* | | | |  |
| **6.1** | to discuss about your life history and development | | | | **1 2 3 4 5** |
| **6.2** | to discuses about your family and social interactions | | | | **1 2 3 4 5** |
| **6.3** | to discuss regarding employment status and financial issues | | | | **1 2 3 4 5** |
| **6.4** | to discuss and assess psychological status | | | | **1 2 3 4 5** |
|  |  | | | |  |
| **Health care organization** | | | | | |
| **1** | Ensuring access, effective and efficient coordination of care | | | |  |
| **1.1** | You travelled longer distance to access this health facility | | | | **1 2 3 4 5** |
| **1.2** | There are reminder notices for specific interventions in the HCO ( how to cover mouth while coughing, hand and mouth care) | | | | **1 2 3 4 5** |
| **1.3** | HCO is designed for comfort and safe to get TB treatment | | | | **1 2 3 4 5** |
| **1.4** | The waiting rooms and other spaces within the premises  are comfortable to follow health education | | | | **1 2 3 4 5** |
|  |  | | | |  |
| **2** | Establishing and strengthening multidisciplinary care teams | | | |  |
|  | Availability of patient support service: | | | |  |
| **2.1** | availing transport | | | | **1 2 3 4 5** |
| **2.2** | food support | | | | **1 2 3 4 5** |
| **2.3** | traditional medicine | | | | **1 2 3 4 5** |
| **2.4** | spiritual | | | | **1 2 3 4 5** |
| **2.5** | Social | | | | **1 2 3 4 5** |
|  | **TB care delivery health system** | | | |  |
| **1** | **Monitoring and addressing patient and community concerns about health care quality** | | | |  |
|  | *TB care service system:* | | | |  |
| **1.1** | You are well transitioned from diagnosis to treatment | | | | **1 2 3 4 5** |
| **1.2** | There is good arrangement of appointments for follow up | | | | **1 2 3 4 5** |
| **1.3** | The TB care service is well integrated with HIV/ART services | | | | **1 2 3 4 5** |
| **1.4** | HCPs made mutual agreements about the care given to you | | | | **1 2 3 4 5** |
| **1.5** | There is a notice with the name and contact details of the person in charge of TB care | | | | **1 2 3 4 5** |
| **1.6** | Your clinical information have been kept confidentially | | | | **1 2 3 4 5** |
|  |  | | | |  |
| **2** | **Teamwork and teambuilding** | | | |  |
|  | *The TB care service providers are:* | | | |  |
| **2.1** | There is good inter-facility referral system | | | | 1 2 3 4 5 |
| **2.2** | There is good intra-facility referral system | | | | 1 2 3 4 5 |
| **2.3** | There is good collaboration among registration, laboratory, treatment and discharge services | | | | 1 2 3 4 5 |
|  |  | | | |  |
| **3** | Assisting people who have experienced adverse events in the health System | | | |  |
| **3.1** | You were highly assisted while you faced difficult situations in the TB care provision | | | | **1 2 3 4 5** |
| **3.2** | There was good compensation system for faced difficult situation, where appropriate | | | | **1 2 3 4 5** |
| **3.3** | fixed contact persons is assigned for questions, problems and complaints | | | | **1 2 3 4 5** |
| **3.4** | There is good reassurance while you faced inconvenience with TB care delivery system | | | | **1 2 3 4 5** |

Telephonic-interview guide :

1. What information the HCPs gave you about TB while you were on treatment?
2. How do you explain the HCPs kept your preference or choice while you were taking your TB treatment?
3. At what extent you took part in your anti TB treatment plan?
4. At what extents HCPs support you to improving your capacity for self-management and self-care with regard to TB?
5. How do you explain the HCPs support, communication, provision of value and respect to patients with TBs to cope with TB treatment?
6. What extent the HCPs discussed with you about bio psychosocial (development, social, financial, psychological) perspectives?
7. How do you explain the overall structure, comfortableness of the health care facility where you diagnosed as you have TB and started treatment?
8. How was the relationship/link among one department with others in health care service organization: registration room, laboratory with treatment room?
9. What was/ were the service/s you used linked with TB treatment?
10. How you were assisted while you faced difficult situations in the TB care provision, if any?
11. In general, how do you explain DOTS service patient centeredness?
12. What do you suggest to reinforce to patient centeredness of DOTS?

FGDs guiding questions:

1. How TB treatment is being provided in Addis Ababa for patients with TB?
2. How patients with TBs select their treatment supporter?
3. How patients with TBs’ preferences are being kept during the treatment?
4. How patient centeredness of DOTS strategy is being explained?
5. What are the measures that can indicate patient centeredness of the DOTS strategy?
6. What are the factors contribute to patient centeredness?
7. Is there anything more you would like to add?
